# Trends of Regional Anesthesia Studies in Emergency Medicine: An Observational Study of Published Articles

**DOI:** 10.5811/westjem.2022.8.57552

**Published:** 2022-10-24

**Authors:** Tou-Yuan Tsai, Hsin-Tzu Yeh, Yu-Chang Liu, Ching-Hsing Lee, Kuan-Fu Chen, Eric H. Chou, Jen-Tang Sun, Kuo-Chih Chen, Yi-Kung Lee, Su Weng Chau

**Affiliations:** * Dalin Tzu Chi Hospital, Buddhist Tzu Chi Medical Foundation, Department of Emergency Medicine, Chiayi, Taiwan; † Tzu Chi University, School of Medicine, Hualien, Taiwan; ‡ Chang Gung Memorial Hospital, Linkou Branch, Department of Emergency Medicine, Taoyuan, Taiwan; § Chi Mei Medical Center, Department of Emergency Medicine, Tainan, Taiwan; ¶ Chang Gung Memorial Hospital, Community Medicine Research Center, Keelung, Taiwan; || Chang Gung University College of Medicine, Chang Gung Memorial Hospital, Department of Emergency Medicine, Keelung, Taiwan; # Chang Gung University, Clinical Informatics and Medical Statistics Research Center, Taoyuan, Taiwan; ** Chang Gung Memorial Hospital, Department of Emergency Medicine, Keelung, Taiwan; †† Baylor Scott & White All Saints Medical Center, Department of Emergency Medicine, Fort Worth, Texas; ‡‡ Baylor University Medical Center, Department of Emergency Medicine, Dallas, Texas; §§ Far Eastern Memorial Hospital, Department of Emergency Medicine, New Taipei City, Taiwan; |||| Taipei Medical University, Shuang Ho Hospital, Department of Emergency Medicine, New Taipei City, Taiwan

## Abstract

**Introduction:**

Regional anesthesia (RA) has become a prominent component of multimodal pain management in emergency medicine (EM), and its use has increased rapidly in recent decades. Nevertheless, there is a paucity of data on how RA practice has evolved in the specialty. In this study we sought to investigate how RA has been implemented in EM by analyzing trends of published articles and to describe the characteristics of the published research.

**Methods:**

We retrieved RA-related publications from the SciVerse Scopus database from inception to January 13, 2022, focusing on studies associated with the use of RA in EM. The primary outcome was an analysis of trend based on the number of annual publications. Other outcomes included reports of technique diversity by year, trends in the use of individual techniques, and characteristics of published articles. We used linear regression analysis to analyze trends.

**Results:**

In total, 133 eligible publications were included. We found that overall 23 techniques have been described and results published in the EM literature. Articles related to RA increased from one article in 1982 to 18 in 2021, and the rate of publication has increased more rapidly since 2016. Reports of lower extremity blocks (60.90%) were published most frequently in ranked-first aggregated citations. The use of thoracic nerve blocks, such as the erector spinae plane block, has increased exponentially in the past three years. The United States (41.35%) has published the most RA-related articles. Regional anesthesia administered by emergency physicians (52.63%) comprised the leading field in published articles related to RA. Most publications discussed single-shot (88.72%) and ultrasound-guided methods (55.64%).

**Conclusion:**

This study highlights that the number of published articles related to regional anesthesia in EM has increased. Although RA research has primarily focused on lower extremity blocks, clinical researchers continue to broaden the field of study to encompass a wide spectrum of techniques and indications.

## INTRODUCTION

The history of regional anesthesia (RA) began with the discovery of the local anesthetic properties of cocaine in 1884.^1^ The first formal pain management organization, the American Society of Regional Anesthesia and Pain Medicine, was founded in 1923 in honor of Gaston Laba, who is considered the “father” of RA and pain medicine.^2^ Research on RA was first published in the emergency medicine (EM) literature in the 1980s with articles describing the use of femoral nerve block to treat femoral fractures.^3^,^4^ Numerous studies have demonstrated that nerve blocks can lower pain scores more than systemic analgesia.^5^,^6^ Using RA can also reduce the incidence of delirium, length of hospital stay, and mortality rate, even when administered in the emergency department (ED) or prehospital setting.^7^,^8^ In EM, indications for RA are diverse, including shoulder reduction, acute pain management in traumatic fracture, headache, herpes zoster, acute pancreatitis, and paraphimosis reduction.^9^–^13^

Despite indications that RA is becoming a universally adopted technique, there is a paucity of data on how RA practice has evolved in EM. Therefore, our primary aim in this study was to investigate the published research trends associated with RA in EM from inception to 2021. Our secondary aim was to describe the characteristics of the published research studies and their content.

## METHODS

### Study Design and Setting

This was a retrospective observational study in which we looked at all the publication and citation data retrieved from the SciVerse Scopus database.^14^ The study protocol was approved by the Institutional Review Board of Chang Gung Medical Foundation, Taiwan (No. 202200609B1).

### Article Selection and Assessment

Regional anesthesia is defined as a specific anesthetic technique that inhibits nerve transmission to avoid or relieve pain, including spinal anesthesia, epidural anesthesia, and nerve blocks.^15^ Emergency medicine encompasses initial evaluation and treatment of any patient requiring expeditious medical and surgical care in a hospital-based or freestanding emergency department (ED), urgent care clinic, or prehospital setting such as an emergency medical response vehicle or a disaster site.^16^ Using the SciVerse Scopus database, we retrieved all publications about RA in EM published up to January 13, 2022. We searched the literature using the following keywords: “regional anesthesia,” “nerve block,” “preoperative,” and “emergency.” We searched for the keywords and linked the search terms with logical Boolean operators in the fields of the title, abstract, and keywords with the type of article.^17^ We excluded studies related to cesarean section, irreversible pulpitis, arthroplasty, arthroscopy, endarterectomy, or herniorrhaphy because of differences in settings, targets, and procedures.

Two reviewers (TYT and HTY) independently screened the titles and abstracts of articles that met the inclusion criteria in the search strategy. Full texts of potentially eligible studies were retrieved and further assessed for eligibility by different reviewers. Inter-reviewer disagreements were resolved by consensus, and a third reviewer (SWC) was consulted, if necessary. Two reviewers independently extracted the following data from the included articles: name of the first author; year of publication; number of citations; country of origin; article categories; publishing journal name; technique described; type of article; site where RA was administered; specialty of the clinician; participants; method of RA guidance; and RA regimens. The country of origin was determined based on the home institution or country of the first author. We further categorized the articles by type, such as case reports, research articles, and reviews, as specified by PubMed. Based on consensus, we categorized the techniques for nerve block into seven fields: upper extremity blocks; lower extremity blocks; thoracic nerve blocks; abdominal nerve blocks; head and neck blocks; cervical plexus blocks; and pudendal and paracervical blocks.^18^,^19^

### Measurements

The primary outcomes were the numbers of research articles published annually and the types of RA techniques described, which we used to evaluate the relationship between the number of papers/techniques and the trend of publication from inception to 2021. We also evaluated papers that we classified according to RA technique categories, which indicate the evolution of the RA research focus in EM. Other measures included the geographic distribution of papers by country, proportion of papers that were published in dedicated EM journals, and distribution of the publication year, type of article, setting, specialty of the RA clinicians, participants, methods of RA guidance, and anesthetic regimens.

### Data Analysis

Because this study encompasses the entirety of available EM citations, rather than a representative sample, we report only descriptive statistics for the distribution of the numbers of article and citations. To analyze trends in the numbers of studies published, we used linear regression analysis. We used the slope (β) of the linear regression curve to represent the trend, and we calculated the 95% confidence intervals (CI) of β. A *P*-value <0.05 was considered statistically significant. We conducted all analyses using STATA, version 17.0 (StataCorp, College Station, TX).

## RESULTS

### Search Results and Regional Anesthesia-related Publication Trends

The initial SciVerse Scopus database search yielded 1,602 articles. We excluded 1,411 papers after screening the titles and abstracts for irrelevant topics, unavailable data, and duplicate records. Another 58 potentially relevant studies were excluded after a full-text review because the word “emergency” appeared in the abstract but RA was not administered in the ED or prehospital setting. After a careful review process, 133 published articles met all eligibility criteria (Supplementary Table S1): 72 (54.14%) articles were categorized as research articles; 50 (37.59%) as case reports; and 11 (8.27%) as reviews. Overall, the total, average, and median numbers of citations were 2,197, 19.44, and 10, respectively.

**Figure 1:**
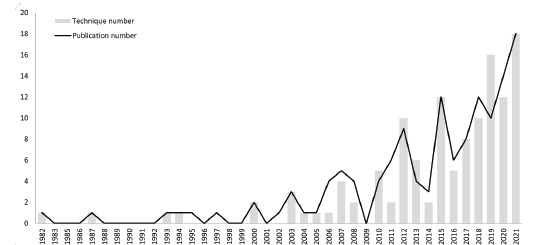
Annual numbers of published articles about techniques for performing regional anesthesia in the emergency department.

The number of RA-related articles in EM increased from one in 1982 to 18 in 2021. The number of articles has been increasing steadily since 1990 and has grown more rapidly since 2016 (Figure 1), with an increasing rate of papers published of 2.17 per year (*P*-value = 0.005, 95% CI 1.08–3.26). The trend in the publication of RA technique types was similar, with an increasing rate of 2.37 per year since 2016 (*P*-value = 0.01, 95% CI 0.85–3.90).

### Characteristics of the Regional Anesthesia-related Papers

The 133 retrieved papers originated from 21 countries, and most studies were from the United States (55/133, 41.35%) (Table 1). Articles were published in 61 journals, with the majority published in the *American Journal of Emergency Medicine* (31/133, 23.31%), followed by the *Journal of Emergency Medicine* (16/133, 12.03%) (Supplementary Table S2). Overall, 118 (88.72%) articles reported that RA was performed in an ED and 13 (9.77%) in the prehospital setting (Table 2). Two included articles surveyed the efficacy of preoperative nerve blocks in acute traumatic hip fracture treatment with outcomes associated with the ED. However, these two studies did not mention the locations where the nerve blocks were performed. We categorized the two studies as “unavailable data” on setting.^20^,^21^ Regional anesthesia was primarily administered by emergency physicians (52.63%) in adult patients (77.44%), and with an ultrasound-guided method (55.64%). Most publications reported use of a single-shot technique (88.72%). Bupivacaine (36.84%) and lidocaine (29.32%) were the local anesthetics most commonly used for injections (Supplementary Table S3).

### Trends in Individualized Regional Anesthesia Techniques

**Figure 2:**
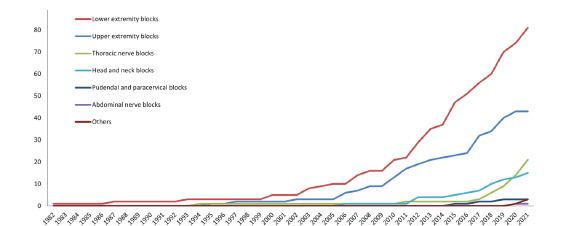
Timeline of published research on regional anesthesia since first being described in 1982. The graph shows an increasing trend in the use of lower extremity blocks, upper extremity blocks, thoracic nerve blocks, and head and neck blocks by emergency physicians.

In total, 23 RA techniques in the seven nerve block categories were described in the retrieved articles (Table 3). The technique most frequently reported in the published EM literature was a femoral nerve block (34/133, 25.56%), which was also the first RA technique reported in EM in 1982. Fascia iliaca compartment block (32/133, 24.06%) ranked second. Of note, the erector spinae plane block (13/133, 9.77%) was the third most published RA technique. The cumulative number of articles annually, sorted according to the seven nerve block categories, showed an upward trend (Figure 2). Lower extremity blocks were the leading research field prior to 2005. Interestingly, after 2016 research on upper extremity blocks, thoracic nerve blocks, and head and neck blocks increased exponentially.

## DISCUSSION

Our study provides a statistical viewpoint of the evolution of publication trends in the use of RA in EM. In this study, we analyzed the publishing trends of RA-related articles using the number of overall papers, technique type, and numbers of citations. The trend in the number of articles and technique types has been increasing since the 1980s and has exponentially increased in the past five years. Most RA-related papers were published in the US. Emergency physician-administered, ultrasound-guided method, single-shot, lower extremity blocks comprised the leading fields in RA-related research. To the best of our knowledge, this is the first study to evaluate the trends in RA-related EM research and analyze their characteristics.

In recent years, the opioid epidemic has emerged as one of the most critical challenges in EM.^22^ As one part of a multimodal analgesic regimen, RA provides a site-specific afferent neural block, achieving timely pain control with fewer complications compared with the administration of opioids. Regional anesthesia is based on the hypothesis that local injection of anesthetic drugs inhibits the propagation of impulses in nerve terminals to inhibit the perception of pain by the cerebral cortex. Evidence has demonstrated that RA could be useful as a tool to decrease the use of opioids for pain in the ED.^23^ Table 3 and Figure 2 show the diverse RA techniques used in EM and the 18 different techniques that were described in published research in 2021.

Regional anesthesia is currently the easiest, fastest, safest, and overall most effective and economical pain-blocking technique, making it the preferred method for emergency cases and patients with many comorbidities or with contraindications to the use of nonsteroidal inflammatory drugs or opioids. It has been used in EM since Berry pioneered its use as a femoral nerve block in femoral fractures in 1977.^4^,^24^ Because a fascia iliaca compartment block provides a more consistent simultaneous blockade of the lateral femoral cutaneous nerves and does not require a nerve stimulator, it has also been widely used for femoral fractures since 2003.^25^ The femoral nerve block and fascia iliaca compartment block were reported in the top three cited papers and most frequently in the top 10 cited papers (Supplementary Table S1).^25^–^27^ Articles about those blocks also steadily increased yearly (Figure 2).

A novel regional technique, the pericapsular nerve group block, has been shown to provide better pain reduction for hip fracture than femoral nerve block.^28^ However, only one case series in EM has been published to date.^29^ Regional anesthesia could also have potential application in shoulder reduction. In fact, one study reported that, unlike procedural sedation, RA shortened the length of ED stay, provided sufficient pain control, and contributed to patient satisfaction in shoulder reduction.^30^ Because of these strengths, articles associated with different RA techniques for shoulder reduction increased in the last decade. Interestingly, articles about the erector spinae plane block were cited often in recent years. A study by Luftig et al demonstrated that the erector spinae plane block is effective for pain control in patients with posterior rib fractures. Their article was published in 2018 and has been cited 50 times to date.^31^ In addition, the erector spinae plane block was reported applicable for pain control in herpes zoster, and a study by Tekin et al has been cited 18 times since its publication in 2019.^11^

While nerve stimulators were used in the past for guidance, more recent evidence has demonstrated that RA performed based on landmarks can be effective and more feasible in a typically busy ED.^26^ A review article revealed that 46% of emergency physicians in the United Kingdom use the landmark-guided femoral nerve block for femoral fractures.^32^ Currently, evidence shows that ultrasound-guided RA provides an increased success rate, shorter procedure time, and fewer complications compared with peripheral nerve stimulation and landmark-guided RA.^33^,^34^ Ultrasound-guided RA is becoming a universally adopted tool.^35^ This trend is comparable with our results. Our study demonstrated that more than half of RA-related articles reported the use of ultrasound-guided methods (55.64%), primarily after 2007 (Table 2).

In our study, nearly 90% of publications reported use of the single-shot block in EM. Compared with the single-shot block, a continuous nerve blockade takes more time and requires more resources in terms of staff, equipment, and capacity. It is not feasible in crowded and busy EDs.^33^ Lidocaine and bupivacaine are the two drugs used in the ED for RA.^3^,^4^,^24^ Both drugs were reported in about one third of publications (Supplementary Table S3). The desired characteristics depend on the patient’s circumstances. A long-acting drug, such as bupivacaine, is desirable for prolonged postoperative analgesia, but it would be particularly problematic in orthopedic assessment after surgery. A short-acting drug, such as lidocaine, is effective for pain control during a radiology examination and transport to the ED, but a bolus is needed after its effects fade.

In the past, many physicians (except anesthesiologists) regarded RA as too complex and intimidating.^36^ One potential barrier is the belief that RA is a technique performed only by anesthesiologists. Another barrier may be the longer time to perform RA than conventional pain management.^37^ However, the implementation of educational and awareness strategies of RA among clinical staff in the ED resulted in a significant increase in the administration of nerve blocks.^38^ Not only emergency physicians, but junior doctors, emergency medical services nurses, and paramedics can improve their competence in basic nerve block procedures with training curricula.^25^,^39^–^41^ Evidence indicates that such staff could safely administer RA with a high success rate and no complications.

A qualitative study surveyed patients about their experience with receiving landmark-based fascia iliaca compartment block performed by paramedics at the scene where the patients suffered their injury.^42^ Interestingly, patients recalled the high quality of care given by paramedics, experienced relief when a fascia iliaca compartment block was given, and had little or no memory of being offered, consenting to, or receiving the block a few weeks after the block was performed. In addition, RA could be performed in other settings, such as disaster sites.^43^ Because of its convenience and effectiveness, we hope that RA can be administrated by more diverse healthcare staff and applied in diverse situations.

## LIMITATIONS

The main strength of this observational study is its complete review of current RA-related publications in EM. However, it also has some limitations. First, the impact of studies from 2022 was underestimated, as no studies published in 2022 were included. This was a result of the gap between the paper’s publication and the appearance of citations in other journals. More recently published studies in 2021 have not had time to accumulate citations. The same phenomenon is mentioned in a previous similar study.^44^ Second, in our study, the published country was sorted according to the affiliation of the first author. Collaborative research between other countries would have been missed because of this methodology. This limitation caused underestimation of collaborative researchers’ contributions.

Third, it is possible that our data could be biased because of the misclassification of the sites where RA was administered and of the specialties of the physicians who performed the procedure. Although our intent was to categorize those variables precisely, the different systems between countries resulted in different definitions of healthcare sites and specialist types. Lastly, there is a potential risk of publication bias. Highly cited topics were based on acceptance of a manuscript with significant results. However, the impact of some topics may be underestimated because of the rare citation or rejection of a manuscript without positive, significant, or interesting results.

## CONCLUSION

This study highlights that the number of articles documenting the use of regional anesthesia in the ED continues to increase. Compared with other techniques, lower extremity block reports were most frequently published in ranked-first aggregated citations.

**Population Health Research Capsule**

What do we already know about this issue?

*Regional anesthesia (RA) has become a prominent component of pain management in the emergency department because it can be a more effective pain reliever than systemic analgesia*.

What was the research question?

*Our goal was to investigate trends in published research associated with RA in emergency medicine (EM) and to describe its characteristics*.

What was the major finding of the study?

*Among 23 techniques in the EM literature, the most common were studies of lower extremity blocks (61% of papers), reported from the US, and described single injections (89%) with ultrasound guidance (56%)*.

How does this improve population health?

*Although RA research has primarily focused on lower extremity blocks, clinical researchers continue to broaden the field of study to encompass a wide spectrum of techniques and indications*.

## Supplementary Information







## Figures and Tables

**Table 1:** Country distribution in regional anesthesia-related publications by the number of publications.

Country	Number of publications	%	Overall times cited
United States	55	41.35	1,072
France	12	9.02	173
United Kingdom	11	8.27	318
Turkey	11	8.27	88
Australia	7	5.26	71
India	6	4.51	41
Iran	6	4.51	19
Netherlands	4	3.01	75
Japan	3	2.26	38
Canada	3	2.26	25
Switzerland	3	2.26	7
Italy	2	1.50	32
Germany	2	1.50	19
Denmark	1	0.75	185
Belgium	1	0.75	17
Hong Kong	1	0.75	11
Tunisia	1	0.75	3
China	1	0.75	1
South Africa	1	0.75	1
Spain	1	0.75	1
Sweden	1	0.75	0

**Table 2:** Characteristics of regional anesthesia-related articles.

	Number of publications*	%
Setting		
ED	118	88.72
Prehospital	13	9.77
NA	2	1.50
Population		
Adults	103	77.44
Pediatrics	12	9.02
Both	7	5.26
NA	11	8.27
Type		
Single	118	88.72
Continuous	3	2.26
Both	1	0.75
NA	11	8.27
Guidance method		
Ultrasound	74	55.64
Landmark	47	35.34
Nerve stimulator	11	8.27
NA	12	9.02
Clinician or other personnel		
Emergency physician	70	52.63
Anesthesiologist	16	12.03
Orthopedist	4	3.01
Paramedic	4	3.01
Radiologist	1	0.75
Neurologist	1	0.75
Pediatrician	1	0.75
ED nurse	1	0.75
Specialist acute pain nurse	1	0.75
NA	44	33.08

* Some articles mentioned more than one characteristic.

ED, emergency department; NA, not available.

**Table 3:** Distribution of individual nerve block techniques reported in regional anesthesia-related articles.

Technique	Number of articles*	%	Overall times cited
Lower extremity block			
Femoral nerve block	34	25.56	580
Fascia iliaca compartment block	32	24.06	837
Sciatic nerve block (transgluteal and popliteal)	11	8.27	67
Superior cluneal nerve block	1	0.75	7
Genicular nerve block	1	0.75	0
Posterior tibial nerve block	2	1.50	7
Total	81	60.90	1,498
Upper extremity block			
Interscalene block	8	6.02	158
Supraclavicular block	6	4.51	99
Infraclavicular block	4	3.01	14
Axillary block	9	6.77	93
Wrist block (radial, ulnar, or median nerve)	13	9.77	168
Suprascapular nerve block	3	2.26	39
Total	43	32.33	571
Thoracic nerve block			
Erector spinae plane block	13	9.77	115
Serratus anterior plane block	6	4.51	53
Intercostal nerve block	1	0.75	20
Interpleural block	1	0.75	23
Total	21	15.79	211
Abdominal nerve block			
Transversus abdominis plane block	1	0.75	1
Total	1	0.75	1
Head and neck block			
Greater occipital nerve block	7	5.26	75
Superficial cervical plexus block	2	1.50	45
Others (orbital, mental, and auricular nerves)	6	4.51	18
Total	15	11.27	138
Pudendal and paracervical block			
Dorsal penile nerve block	3	2.26	13
Total	3	2.26	13
Other			
Stellate ganglion block	2	1.50	3
Spinal accessory nerve block	1	0.75	0

* Some articles mentioned more than one technique.
