# Supplementary material for: Trends of Regional Anesthesia Studies in Emergency Medicine: An Observational Study of Published Articles
Source: West J Emerg Med. 2022 Oct 24;23(6):878–85. doi: 10.5811/westjem.2022.8.57552 (PMC9683772; doi:10.5811/westjem.2022.8.57552)
Supplement: Supplementary file 1 [file wjem-23-878-s001.docx]

**Supplementary Table S1.** Regional anesthesia-related publications in emergency medicine by the number of citations.

| **No.** | **Title** | **First**  **author** | **Country** | **Journal name** | **Year** | **Overall cited times** |
| --- | --- | --- | --- | --- | --- | --- |
| 1 | Fascia iliaca compartment blockade for acute pain control in hip fracture patients: A randomized, placebo-controlled trial | Foss N.B. | Denmark | Anesthesiology | 2007 | 185 |
| 2 | Three-in-one femoral nerve block as analgesia for fractured neck of femur in the emergency department: A randomized, controlled trial | Fletcher A.K. | United Kingdom | Annals of Emergency Medicine | 2003 | 127 |
| 3 | Fascia iliaca compartment block for femoral bone fractures in prehospital care | Lopez S. | France | Regional Anesthesia and Pain Medicine | 2003 | 105 |
| 4 | A Randomized Controlled Trial Comparing a Fascia Iliaca Compartment Nerve Block to a Traditional Systemic Analgesic for Femur Fractures in a Pediatric Emergency Department | Wathen J.E. | United States | Annals of Emergency Medicine | 2007 | 81 |
| 5 | Ultrasound-guided femoral nerve blocks in elderly patients with hip fractures | Beaudoin F.L. | United States | American Journal of Emergency Medicine | 2010 | 73 |
| 6 | Feasibility of Forearm Ultrasonography-Guided Nerve Blocks of the Radial, Ulnar, and Median Nerves for Hand Procedures in the Emergency Department | Liebmann O. | United States | Annals of Emergency Medicine | 2006 | 70 |
| 7 | A comparison of ultrasound-guided three-in-one femoral nerve block versus parenteral opioids alone for analgesia in emergency department patients with hip fractures: A randomized controlled trial | Beaudoin F.L. | United States | Academic Emergency Medicine | 2013 | 65 |
| 8 | Ultrasound-guided fascia iliaca compartment block for hip fractures in the emergency department | Haines L. | United States | Journal of Emergency Medicine | 2012 | 63 |
| 9 | A prospective comparison of procedural sedation and ultrasound-guided interscalene nerve block for shoulder reduction in the emergency department | Blaivas M. | United States | Academic Emergency Medicine | 2011 | 63 |
| 10 | Ultrasound-guided supraclavicular brachial plexus nerve block vs procedural sedation for the treatment of upper extremity emergencies | Stone M.B. | United States | American Journal of Emergency Medicine | 2008 | 56 |
| 11 | Successful emergency pain control for posterior rib fractures with ultrasound-guided erector spinae plane block | Luftig J. | United States | American Journal of Emergency Medicine | 2018 | 50 |
| 12 | Ultrasound-guided serratus plane block for ED rib fracture pain control | Durant E. | United States | American Journal of Emergency Medicine | 2017 | 47 |
| 13 | A comparison of pre-operative nerve stimulator-guided femoral nerve block and fascia iliaca compartment block in patients with a femoral neck fracture | Newman B. | United Kingdom | Anaesthesia | 2013 | 47 |
| 14 | The ultrasound-guided superficial cervical plexus block for anesthesia and analgesia in emergency care settings | Herring A.A. | United States | American Journal of Emergency Medicine | 2012 | 44 |
| 15 | Paramedic-performed fascia iliaca compartment block for femoral fractures: A controlled trial | McRae P.J. | Australia | Journal of Emergency Medicine | 2015 | 42 |
| 16 | Prehospital administered fascia iliaca compartment block by emergency medical service nurses, a feasibility study | Dochez E. | Netherlands | Scandinavian Journal of Trauma, Resuscitation and Emergency Medicine | 2014 | 41 |
| 17 | Femoral nerve block in the initial management of femoral shaft fractures | McGlone R. | United Kingdom | Archives of Emergency Medicine | 1987 | 39 |
| 18 | Ultrasound-guided interscalene block for shoulder dislocation reduction in the ED | Blaivas M. | United States | American Journal of Emergency Medicine | 2006 | 38 |
| 19 | Femoral nerve block for diaphyseal and distal femoral fractures in the emergency department | Mutty C.E. | United States | Journal of Bone and Joint Surgery - Series A | 2007 | 37 |
| 20 | Fascia iliaca block for femoral bone fractures in prehospital medicine [Bloc iliofascial en médecine préhospitalière pour les fractures du fémur] | Gozlan C. | France | Annales Francaises d'Anesthesie et de Reanimation | 2005 | 31 |
| 21 | Femoral nerve block for fractured shaft of femur | Tondare A.S. | India | Canadian Anaesthetists' Society Journal | 1982 | 31 |
| 22 | Ultrasound-guided supraclavicular block for the treatment of upper extremity fractures, dislocations, and abscesses in the ED | Stone M.B. | United States | American Journal of Emergency Medicine | 2007 | 30 |
| 23 | Axillary block for analgesia during manipulation of forearm fractures in the pediatric emergency department: A prospective randomized comparative trial | Kriwanek K.L. | United States | Journal of Pediatric Orthopaedics | 2006 | 26 |
| 24 | Single fascia iliaca compartment block is safe and effective for emergency pain relief in hip-fracture patients | Groot L. | Netherlands | Western Journal of Emergency Medicine | 2015 | 24 |
| 25 | A comparison of suprascapular nerve block and procedural sedation analgesia in shoulder dislocation reduction | Tezel O. | Turkey | American Journal of Emergency Medicine | 2014 | 24 |
| 26 | Great occipital nerve blockade for cluster headache in the emergency department: Case report | Scattoni L. | Italy | Journal of Headache and Pain | 2006 | 23 |
| 27 | Reduction of forearm fractures in children using axillary block anesthesia | Cramer K.E. | United States | Journal of Orthopaedic Trauma | 1995 | 23 |
| 28 | Interpleural block for patients with multiple rib fractures: Comparison with epidural block | Shinohara K. | Japan | Journal of Emergency Medicine | 1994 | 23 |
| 29 | Ultrasound-guided nerve blocks in emergency medicine practice | Amini R. | United States | Journal of Ultrasound in Medicine | 2016 | 22 |
| 30 | Randomised trial of the fascia iliaca block versus the '3-in-1' block for femoral neck fractures in the emergency department | Reavley P. | United Kingdom | Emergency Medicine Journal | 2015 | 22 |
| 31 | Pain control in disaster settings: A role for ultrasound-guided nerve blocks | Lippert S.C. | United States | Annals of Emergency Medicine | 2013 | 22 |
| 32 | Femoral nerve block for femoral shaft fractures in a paediatric emergency department: Can it be done better? | Chu R.S.L. | Australia | European Journal of Emergency Medicine | 2003 | 21 |
| 33 | Ultrasound-guided intercostal nerve block for traumatic pneumothorax requiring tube thoracostomy | Stone M.B. | United States | American Journal of Emergency Medicine | 2011 | 20 |
| 34 | Feasibility and safety of ultrasound-guided nerve block for management of limb injuries by emergency care physicians | Bhoi S. | United States | Journal of Emergencies, Trauma and Shock | 2012 | 19 |
| 35 | Peridural Anesthesia or Ultrasound-Guided Continuous 3-in-1 Block: Which Is Indicated for Analgesia in Very Elderly Patients With Hip Fracture in the Emergency Department? | Luger T.J. | Germany | Geriatric Orthopaedic Surgery & Rehabilitation | 2012 | 19 |
| 36 | High-thoracic ultrasound-guided erector spinae plane block for acute herpes zoster pain management in emergency department | Tekin E. | Turkey | American Journal of Emergency Medicine | 2019 | 18 |
| 37 | The effectiveness of greater occipital nerve blockade in treating acute migraine-related headaches in emergency departments | Korucu O. | Turkey | Acta Neurologica Scandinavica | 2018 | 18 |
| 38 | Fascia iliaca compartment block for hip fractures: Experience of integrating a new protocol across two hospital sites | Lees D. | United Kingdom | European Journal of Emergency Medicine | 2016 | 18 |
| 39 | Impact of ultrasound-guided femoral nerve blocks in the pediatric emergency department | Turner A.L. | United States | Pediatric Emergency Care | 2014 | 18 |
| 40 | Ultrasound-guided femoral nerve block for pain control in an infant with a femur fracture due to nonaccidental trauma | Frenkel O. | Canada | Pediatric Emergency Care | 2012 | 18 |
| 41 | Ultrasound-guided interscalene nerve block vs procedural sedation by propofol and fentanyl for anterior shoulder dislocations | Raeyat Doost E. | Iran | American Journal of Emergency Medicine | 2017 | 17 |
| 42 | Standard preoperative analgesia with or without fascia iliaca compartment block for femoral neck fractures | Williams H. | United Kingdom | Journal of Orthopaedic Surgery | 2016 | 17 |
| 43 | Emergency department use of a continuous femoral nerve block for pain relief for fractured femur in children | Stewart B. | United Kingdom | Emergency Medicine Journal | 2007 | 17 |
| 44 | The 'three in one' block as locoregional analgesia in an emergency department. | Van Leeuwen F.L. | Belgium | European journal of emergency medicine : official journal of the European Society for Emergency Medicine | 2000 | 17 |
| 45 | Geriatric trauma G-60 falls with hip fractures: A pilot study of acute pain management using femoral nerve fascia iliac blocks | Mangram A.J. | United States | Journal of Trauma and Acute Care Surgery | 2015 | 16 |
| 46 | Ultrasound-guided suprascapular nerve block for shoulder reduction and adhesive capsulitis in the ED | Herring A.A. | United States | American Journal of Emergency Medicine | 2011 | 16 |
| 47 | Fascia iliaca compartment block reduces morphine requirement pre-operatively for patients with fractured neck of femur | Leeper A.D. | United Kingdom | European Journal of Trauma and Emergency Surgery | 2012 | 15 |
| 48 | Ultrasound-guided Greater Auricular Nerve Block for Emergency Department Ear Laceration and Ear Abscess Drainage | Flores S. | United States | Journal of Emergency Medicine | 2016 | 14 |
| 49 | Femoral nerve block for diaphyseal and distal femoral fractures in the emergency department: Surgical technique | Mutty C.E. | United States | Journal of Bone and Joint Surgery - Series A | 2008 | 13 |
| 50 | A Randomized, Sham-Controlled Trial of Bilateral Greater Occipital Nerve Blocks With Bupivacaine for Acute Migraine Patients Refractory to Standard Emergency Department Treatment With Metoclopramide | Friedman B.W. | United States | Headache | 2018 | 12 |
| 51 | Ultrasound-guided nerve blocks in the emergency department | Bhoi S. | United States | Journal of Emergencies, Trauma and Shock | 2010 | 12 |
| 52 | Erector spinae plane block for bilateral lumbar transverse process fracture in emergency department: A new indication | Ahiskalioglu A. | Turkey | American Journal of Emergency Medicine | 2018 | 11 |
| 53 | Ultrasound-guided dorsal penile nerve block for ED paraphimosis reduction | Flores S. | United States | American Journal of Emergency Medicine | 2015 | 11 |
| 54 | Occipital nerve blocks in the treatment of headaches: Safety and efficacy | Voigt C.L. | United States | Journal of Emergency Medicine | 2015 | 11 |
| 55 | Prehospital analgesia with femoral nerve block following lower extremity injury. A 107 cases survey [Bloc fémoral en analgésie préhospitalière pour traumatisme du membre inférieur. Enquête de pratique observationnelle sur 107 cas] | Gros T. | France | Annales Francaises d'Anesthesie et de Reanimation | 2012 | 11 |
| 56 | A pilot randomised clinical trial of 3-in-1 femoral nerve block and intravenous morphine as primary analgesia for patients presenting to the emergency department with fractured hip | Graham C.A. | Hong Kong | Hong Kong Journal of Emergency Medicine | 2008 | 11 |
| 57 | Relief of refractory renal colic in emergency department: A novel indication for ultrasound guided erector spinae plane block | Aydin M.E. | Turkey | American Journal of Emergency Medicine | 2019 | 10 |
| 58 | Ultrasound-guided retroclavicular approach infraclavicular brachial plexus block for upper extremity emergency procedures | Luftig J. | United States | American Journal of Emergency Medicine | 2017 | 10 |
| 59 | Ultrasound-Guided Femoral Nerve Blocks | Baker M.D. | United States | Pediatric Emergency Care | 2015 | 10 |
| 60 | Supracondylar radial nerve block for treatment of distal radius fractures in the emergency department | Frenkel O. | United States | Journal of Emergency Medicine | 2011 | 10 |
| 61 | Axillary brachial plexus block - An underused technique in the accident and emergency department | Mackay C.A. | United Kingdom | Emergency Medicine Journal | 1997 | 10 |
| 62 | Fascia-iliaca compartment block vs intra-articular hip injection for preoperative pain management in intracapsular hip fractures: A blind, randomized, controlled trial | Aprato A. | Italy | Injury | 2018 | 9 |
| 63 | Ultrasound-guided peripheral forearm nerve block for digit fractures in a pediatric emergency department | Mori T. | Japan | American Journal of Emergency Medicine | 2019 | 8 |
| 64 | Superior Cluneal Nerve Block for Treatment of Buttock Abscesses in the Emergency Department | Herring A. | United States | Journal of Emergency Medicine | 2010 | 8 |
| 65 | A Randomized, Double-Dummy, Emergency Department-Based Study of Greater Occipital Nerve Block With Bupivacaine vs Intravenous Metoclopramide for Treatment of Migraine | Friedman B.W. | United States | Headache | 2020 | 7 |
| 66 | Successful emergency pain control for acute pancreatitis with ultrasound guided erector spinae plane blocks | Mantuani D. | United States | American Journal of Emergency Medicine | 2020 | 7 |
| 67 | The Use of the Erector Spinae Plane Block to Decrease Pain and Opioid Consumption in the Emergency Department: A Literature Review | Abdelhamid K. | Canada | Journal of Emergency Medicine | 2020 | 7 |
| 68 | Fascia iliaca block in the emergency department for hip fracture: A randomized, controlled, double-blind trial | Pasquier M. | Switzerland | BMC Geriatrics | 2019 | 7 |
| 69 | Emergency physician-performed ultrasound-guided nerve blocks in proximal femoral fractures provide safe and effective pain relief: a prospective observational study in The Netherlands | Ketelaars R. | Netherlands | International Journal of Emergency Medicine | 2018 | 7 |
| 70 | Continuous erector spinae plane block for pain management of an extensive burn | Ueshima H. | Japan | American Journal of Emergency Medicine | 2018 | 7 |
| 71 | Emergency Department Pain Management Following Implementation of a Geriatric Hip Fracture Program | Casey S.D. | United States | Western Journal of Emergency Medicine | 2017 | 7 |
| 72 | ED ultrasound-guided posterior tibial nerve blocks for calcaneal fracture analagesia | Clattenburg E. | United States | American Journal of Emergency Medicine | 2016 | 7 |
| 73 | Ultrasound-guided axillary nerve block for ED incision and drainage of deltoid abscess | Lyons C. | United States | American Journal of Emergency Medicine | 2017 | 6 |
| 74 | Sciatic nerve block in prehospital care [Bloc sciatique en médecine préhospitalière] | Gros T. | France | Annales Francaises d'Anesthesie et de Reanimation | 2010 | 6 |
| 75 | The sciatic nerve block in emergency settings: A comparison between a new anterior and the classic lateral approaches | Fuzier R. | France | Medical Science Monitor | 2004 | 6 |
| 76 | Femoral nerve block for acute pain relief in fracture shaft femur in an emergency ward | Somvanshi M. | India | Saudi Journal of Anaesthesia | 2015 | 5 |
| 77 | Interscalene brachial plexus block for glenohumeral luxation in prehospital medicine [Bloc interscalénique pour luxation glénohumérale en médecine préhospitalière] | Lagrabette J.-F. | France | Annales Francaises d'Anesthesie et de Reanimation | 2008 | 5 |
| 78 | Rapid Analgesia for Prehospital hip Disruption (RAPID): Findings from a randomised feasibility study | Jones J.K. | United Kingdom | Pilot and Feasibility Studies | 2019 | 4 |
| 79 | Multitroncular block at the elbow for a major hand trauma, in prehospital care [Blocs tronculaires au pli du coude en urgence prehospitalière] | Lopez S. | France | Annales Francaises d'Anesthesie et de Reanimation | 2002 | 4 |
| 80 | Nerve stimulator-assisted femoral nerve block in the emergency department | Stella J. | Australia | Emergency Medicine | 2000 | 4 |
| 81 | Erector spinae plane block for multiple rib fracture done by an Emergency Physician: A case series | Kumar G. | India | Australasian Journal of Ultrasound in Medicine | 2021 | 3 |
| 82 | Stellate Ganglion Nerve Block by Point-of-Care Ultrasonography for Treatment of Refractory Infarction-Induced Ventricular Fibrillation | Margus C. | United States | Annals of Emergency Medicine | 2020 | 3 |
| 83 | Ultrasound guided supra-inguinal Fascia Iliaca Compartment Blocks in hip fracture patients: An alternative technique | Ridderikhof M.L. | Netherlands | American Journal of Emergency Medicine | 2020 | 3 |
| 84 | Utilizing Ultrasound-Guided Femoral Nerve Blocks and Fascia Iliaca Compartment Blocks for Proximal Femur Fractures in the Emergency Department | Wilbeck J. | United States | Advanced Emergency Nursing Journal | 2019 | 3 |
| 85 | Management of an Acute Exacerbation of Chronic Neuropathic Pain in the Emergency Department: A Case to Support Ultrasound-Guided Forearm Nerve Blocks | Canders C.P. | United States | Journal of Emergency Medicine | 2018 | 3 |
| 86 | Use of occipital nerve block in emergency department treatment of status migrainosus | Yanuck J. | United States | American Journal of Emergency Medicine | 2018 | 3 |
| 87 | Magnesium Sulfate as Adjuvant in Prehospital Femoral Nerve Block for a Patient with Diaphysial Femoral Fracture: A Randomized Controlled Trial | Jebali C. | Tunisia | Pain Research and Management | 2018 | 3 |
| 88 | Ultrasound-Guided Femoral Nerve Blockage in A Patellar Dislocation: An Effective Technique for Emergency Physicians | Eksert S. | Turkey | Journal of Emergency Medicine | 2017 | 3 |
| 89 | Ultrasound-Guided Femoral Nerve Blocks in the Initial Emergency Department Management of Pediatric Femur Fractures | Cross K.P. | United States | Clinical Pediatric Emergency Medicine | 2016 | 3 |
| 90 | Loco-regional anaesthesia in prehospital emergency situations: Fascia iliaca block [Anesthésie locorégionale préhospitalière: Le bloc iliofascial] | Lefort H. | France | Annales Francaises de Medecine d'Urgence | 2013 | 3 |
| 91 | Nerve stimulator-assisted sciatic nerve block for painful procedures in the ED | Phillips W.J. | United States | American Journal of Emergency Medicine | 2011 | 3 |
| 92 | Ultrasound-guided transgluteal sciatic nerve analgesia for refractory back pain in the ED: A case series | Goldsmith A.J. | United States | American Journal of Emergency Medicine | 2020 | 2 |
| 93 | Feasibility and initial experience with continuous nerve blocks by emergency physicians | Martel M.L. | United States | American Journal of Emergency Medicine | 2020 | 2 |
| 94 | Is fascia iliaca compartment block administered by paramedics for suspected hip fracture acceptable to patients? A qualitative study | Evans B.A. | United Kingdom | BMJ Open | 2019 | 2 |
| 95 | Case Report: Ultrasound-Guided Infraclavicular Brachial Plexus Block for a Case with Posterior Elbow Dislocation | Akay S. | Turkey | Journal of Emergency Medicine | 2017 | 2 |
| 96 | Femoral nerve blocks for fractured neck of femur patients: A 'feel good solution' but a 'short-term fix'? | Clothier V. | Australia | EMA - Emergency Medicine Australasia | 2015 | 2 |
| 97 | Urgent interscalene brachial plexus block for management of traumatic luxatio erecta in the ED | Brant-Zawadzki G. | United States | American Journal of Emergency Medicine | 2015 | 2 |
| 98 | Comparison of femoral nerve block with intravenous morphine sulfate for pain relief of femoral fracture | Forouzan A. | Iran | Asian Journal of Scientific Research | 2015 | 2 |
| 99 | Ultrasound-guided supra-inguinal fascia Iliaca compartment block for older adults admitted to the emergency department with hip fracture: a randomized controlled, double-blind clinical trial | Chen L. | China | BMC Geriatrics | 2021 | 1 |
| 100 | Erector spinae plane block: A new option for managing acute axial low back pain in the emergency department | Anshus A.J. | United States | Pain Management | 2021 | 1 |
| 101 | Regional anaesthesia for rib fractures: A pilot study of serratus anterior plane block | Schnekenburger M. | Australia | EMA - Emergency Medicine Australasia | 2021 | 1 |
| 102 | Comparison of greater occipital nerve and supra orbital nerve blocks methods in the treatment of acute migraine attack: A randomized double-blind controlled trial | Hokenek N.M. | Turkey | Clinical Neurology and Neurosurgery | 2021 | 1 |
| 103 | Erector spinae plane block for pain control in patients with pancreatitis in the emergency department | Gopinath B. | India | Turkish Journal of Emergency Medicine | 2021 | 1 |
| 104 | Transversus abdominis plane block: A new method in renal colic pain management | Kadioglu E. | Turkey | American Journal of Emergency Medicine | 2020 | 1 |
| 105 | Ultrasound-Guided Serratus Anterior Plane Block for Intractable Herpes Zoster Pain in the Emergency Department | Goldsmith A.J. | United States | Journal of Emergency Medicine | 2020 | 1 |
| 106 | Ultrasound-guided serratus anterior plane block for rib fracture-associated pain management in emergency department | Paul S. | India | Journal of Emergencies, Trauma and Shock | 2020 | 1 |
| 107 | Nervous breakdown! A registry of nerve blocks from a South African emergency centre | Snyman J. | South Africa | African Journal of Emergency Medicine | 2019 | 1 |
| 108 | Multimodal analgesia in crotalid snakebite envenomation: A novel use of femoral nerve block | Barton D.J. | United States | American Journal of Emergency Medicine | 2018 | 1 |
| 109 | Regional Anesthesia in the Pediatric Emergency Department | Wathen J. | United States | Clinical Pediatric Emergency Medicine | 2017 | 1 |
| 110 | Regional anesthesia of the face in emergency department (part 2): Infra orbital and mental block [Anesthésie locorégionale de la face aux urgences (partie 2): Blocs infra-orbitaire et mentonnier] | Lacroix G. | France | Annales Francaises de Medecine d'Urgence | 2012 | 1 |
| 111 | Regional anesthesia of the face in emergency department: Supraorbital and supratrochlear block [Anesthésie locorégionale de la face aux urgences: Blocs supra-orbitaire et supra-trochléaire] | Lacroix G. | France | Annales Francaises de Medecine d'Urgence | 2012 | 1 |
| 112 | Experience with regional anesthesia for reduction of shoulder dislocation in the emergency department [Experiencia en el empleo de anestesia regional para la reducción de las luxaciones de hombro en urgencias] | Belinchón de Diego E. | Spain | Emergencias | 2011 | 1 |
| 113 | Reduction of acute patellar dislocation under femoral nerve blockade | Hoy G. | Australia | Emergency Medicine | 1993 | 1 |
| 114 | Can emergency nurses safely and effectively insert fascia iliaca blocks in patients with a fractured neck of femur? A prospective cohort study in an Australian emergency department | Gawthorne J. | Australia | Journal of Clinical Nursing | 2021 | 0 |
| 115 | The success rate of posterior tibial nerve block in the ankle with and without ultrasound guidance: a clinical trial study for pain management in emergency departments | Kakhki B.R. | Iran | Journal of Emergency Practice and Trauma | 2021 | 0 |
| 116 | Immediate ultrasound-guided femoral nerve block in proximal femur fractures in the emergency department [Sofortige ultraschallgestützte Femoralisblockade bei proximalen Femurfrakturen in der Notaufnahme] | Schöll E. | Switzerland | Notfall und Rettungsmedizin | 2021 | 0 |
| 117 | Efficacy of fascia iliaca nerve block in daily routine for children with femoral fractures in a pediatric emergency department | Curtis W. | France | Archives de Pediatrie | 2021 | 0 |
| 118 | Novel Use of 3-Point Genicular Nerve Block for Acute Knee Pain in the Emergency Department | Sobel J. | United States | Journal of Emergency Medicine | 2021 | 0 |
| 119 | Erector spinae plane block in the emergency department for upper extremity: A case report | Lee D.H. | United States | Clinical Practice and Cases in Emergency Medicine | 2021 | 0 |
| 120 | Fascia iliaca compartment block (FICB) as pain treatment in older persons with suspected hip fractures in prehospital emergency care – A comparative pilot study | Wennberg P. | Sweden | International Emergency Nursing | 2021 | 0 |
| 121 | Calming the storm – Stellate ganglion block in refractory ventricular arrhythmia in the emergency department | Mohd. Y. | India | American Journal of Emergency Medicine | 2021 | 0 |
| 122 | Successful Ultrasound-Guided Erector Spinae Plane Block for Herpes Zoster in the Emergency Department: A Case Report | Dilip M. | United States | Journal of Emergency Medicine | 2021 | 0 |
| 123 | Ultrasound-Guided Transgluteal Sciatic Nerve Block for Gluteal Procedural Analgesia | Selame L.A. | United States | Journal of Emergency Medicine | 2021 | 0 |
| 124 | The erector spinae plane block for acute pain management in emergency department patients with rib fractures | Surdhar I. | Canada | Canadian Journal of Emergency Medicine | 2021 | 0 |
| 125 | Ultrasound-guided spinal accessory nerve block for intractable trapezius pain: A case series | Herbst M.K. | United States | American Journal of Emergency Medicine | 2021 | 0 |
| 126 | Ultrasound-guided regional anaesthesia and reduction of distal radius fractures in an emergency department | Ammann S. | Switzerland | Swiss medical weekly | 2020 | 0 |
| 127 | Outpatient treatment of acute injuries of upper extremities with axillary plexus anesthesia in the emergency department—Is that possible without continuous anesthesia attendance? [Ambulante Versorgung akuter Verletzungen der oberen Extremität in der Notfallambulanz in axillärer Plexusanästhesie – Ist das ohne durchgehende Anästhesiebegleitung machbar?] | Rand A. | Germany | Anaesthesist | 2020 | 0 |
| 128 | The Feasibility of the Ultrasound-Guided Femoral Nerve Block Procedure with Low-Dose Local Anesthetic in Intracapsular and Extracapsular Hip Fractures | Topal F.E. | Turkey | Journal of Emergency Medicine | 2020 | 0 |
| 129 | Ultrasound-guided supracondylar radial nerve block to manage distal radius fractures in the emergency department | Isfahani M.N. | Iran | Journal of Emergency Medicine, Trauma and Acute Care | 2020 | 0 |
| 130 | Nerve stimulator versus ultrasound-guided femoral nerve block; a randomized clinical trial | Forouzan A. | Iran | Archives of Academic Emergency Medicine | 2019 | 0 |
| 131 | Pain management via ultrasound-guided nerve block in emergency department; A case series study | Nejat A. | Iran | Archives of Academic Emergency Medicine | 2019 | 0 |
| 132 | Effectiveness of regional anaesthesia for treatment of facial and hand wounds by emergency physicians: A 9-month prospective study | Siaffa R. | France | Anaesthesia Critical Care and Pain Medicine | 2018 | 0 |
| 133 | Fascia iliaca compartment block in dislocated hip reduction | Kaldirim U. | Turkey | African Journal of Emergency Medicine | 2015 | 0 |
