# Supplementary material for: Trends of Regional Anesthesia Studies in Emergency Medicine: An Observational Study of Published Articles
Source: West J Emerg Med. 2022 Oct 24;23(6):878–85. doi: 10.5811/westjem.2022.8.57552 (PMC9683772; doi:10.5811/westjem.2022.8.57552)
Supplement: Supplementary file 2 [file wjem-23-878-s002.docx]

**Supplementary Table S2.** Journals that published reginal anesthesia-related publications in emergency medicine by the number of publications.

| **Journal name** | **Number of publications** | **%** | **Overall times cited** |
| --- | --- | --- | --- |
| *American Journal of Emergency Medicine* | 31 | 23.31 | 527 |
| *Journal of Emergency Medicine* | 16 | 12.03 | 187 |
| *Annals of Emergency Medicine* | 5 | 3.76 | 303 |
| *Annales Francaises d'Anesthesie et de Reanimation* | 5 | 3.76 | 57 |
| *Emergency Medicine Journal* | 3 | 2.26 | 49 |
| *Pediatric Emergency Care* | 3 | 2.26 | 46 |
| *Journal of Emergencies, Trauma and Shock* | 3 | 2.26 | 32 |
| *Annales Francaises de Medecine d'Urgence* | 3 | 2.26 | 5 |
| *Academic Emergency Medicine* | 2 | 1.50 | 128 |
| *Journal of Bone and Joint Surgery - Series A* | 2 | 1.50 | 50 |
| *European Journal of Emergency Medicine* | 2 | 1.50 | 39 |
| *Western Journal of Emergency Medicine* | 2 | 1.50 | 31 |
| *Headache* | 2 | 1.50 | 19 |
| *BMC Geriatrics* | 2 | 1.50 | 8 |
| *Emergency Medicine* | 2 | 1.50 | 5 |
| *Clinical Pediatric Emergency Medicine* | 2 | 1.50 | 4 |
| *EMA - Emergency Medicine Australasia* | 2 | 1.50 | 3 |
| *African Journal of Emergency Medicine* | 2 | 1.50 | 1 |
| *Archives of Academic Emergency Medicine* | 2 | 1.50 | 0 |
| *Anesthesiology* | 1 | 0.75 | 185 |
| *Regional Anesthesia and Pain Medicine* | 1 | 0.75 | 105 |
| *Anaesthesia* | 1 | 0.75 | 47 |
| *Scandinavian Journal of Trauma, Resuscitation and Emergency Medicine* | 1 | 0.75 | 41 |
| *Archives of Emergency Medicine* | 1 | 0.75 | 39 |
| *Canadian Anaesthetists' Society Journal* | 1 | 0.75 | 31 |
| *Journal of Pediatric Orthopaedics* | 1 | 0.75 | 26 |
| *Journal of Headache and Pain* | 1 | 0.75 | 23 |
| *Journal of Orthopaedic Trauma* | 1 | 0.75 | 23 |
| *Journal of Ultrasound in Medicine* | 1 | 0.75 | 22 |
| *Geriatric Orthopaedic Surgery & Rehabilitation* | 1 | 0.75 | 19 |
| *Acta Neurologica Scandinavica* | 1 | 0.75 | 18 |
| *European Journal of Emergency Medicine: Official Journal of the European Society for Emergency Medicine* | 1 | 0.75 | 17 |
| *Journal of Orthopaedic Surgery* | 1 | 0.75 | 17 |
| *Journal of Trauma and Acute Care Surgery* | 1 | 0.75 | 16 |
| *European Journal of Trauma and Emergency Surgery* | 1 | 0.75 | 15 |
| *Hong Kong Journal of Emergency Medicine* | 1 | 0.75 | 11 |
| *Injury* | 1 | 0.75 | 9 |
| *International Journal of Emergency Medicine* | 1 | 0.75 | 7 |
| *Medical Science Monitor* | 1 | 0.75 | 6 |
| *Saudi Journal of Anaesthesia* | 1 | 0.75 | 5 |
| *Pilot and Feasibility Studies* | 1 | 0.75 | 4 |
| *Advanced Emergency Nursing Journal* | 1 | 0.75 | 3 |
| *Australasian Journal of Ultrasound in Medicine* | 1 | 0.75 | 3 |
| *Pain Research and Management* | 1 | 0.75 | 3 |
| *Asian Journal of Scientific Research* | 1 | 0.75 | 2 |
| *BMJ Open* | 1 | 0.75 | 2 |
| *Clinical Neurology and Neurosurgery* | 1 | 0.75 | 1 |
| *Emergencias* | 1 | 0.75 | 1 |
| *Pain Management* | 1 | 0.75 | 1 |
| *Turkish Journal of Emergency Medicine* | 1 | 0.75 | 1 |
| *Anaesthesia Critical Care and Pain Medicine* | 1 | 0.75 | 0 |
| *Anaesthesist* | 1 | 0.75 | 0 |
| *Archives de Pediatrie* | 1 | 0.75 | 0 |
| *Canadian Journal of Emergency Medicine* | 1 | 0.75 | 0 |
| *Clinical Practice and Cases in Emergency Medicine* | 1 | 0.75 | 0 |
| *International Emergency Nursing* | 1 | 0.75 | 0 |
| *Journal of Clinical Nursing* | 1 | 0.75 | 0 |
| *Journal of Emergency Medicine, Trauma and Acute Care* | 1 | 0.75 | 0 |
| *Journal of Emergency Practice and Trauma* | 1 | 0.75 | 0 |
| *Notfall und Rettungsmedizin* | 1 | 0.75 | 0 |
| *Swiss Medical Weekly* | 1 | 0.75 | 0 |
