# Supplementary material for: Trends of Regional Anesthesia Studies in Emergency Medicine: An Observational Study of Published Articles
Source: West J Emerg Med. 2022 Oct 24;23(6):878–85. doi: 10.5811/westjem.2022.8.57552 (PMC9683772; doi:10.5811/westjem.2022.8.57552)
Supplement: Supplementary file 3 [file wjem-23-878-s003.docx]

**Supplementary Table S3.** Regimens selected in regional anesthesia-related publications.

| **Regimen** | **Number of publications*** | **%*** |
| --- | --- | --- |
| Bupivacaine | 49 | 36.84 |
| Lidocaine | 39 | 29.32 |
| Ropivacaine | 18 | 13.53 |
| Levobupivacaine | 8 | 6.02 |
| Prilocaine | 6 | 4.51 |
| Mepivacaine | 5 | 3.76 |
| Chloroprocaine | 1 | 0.75 |
| NA | 21 | 15.79 |

*Some articles mentioned more than one regimen.

NA: not available
